# Acute rupture of a huge liver hydatid cysts in the peritoneal cavity causing an anaphylactic shock: A case report

**DOI:** 10.1016/j.radcr.2025.01.098

**Published:** 2025-03-08

**Authors:** Mohamed Zayati, Mohamed Ali Chaouch, Salem Mokni, Mohamed Maaref, Mehdi Abdelwahed, Hafeth Daly, Asma Ladib, Fethi Jebali, Aymen Kawech

**Affiliations:** a Department of Surgery, Monastir University Hospital, Monastir, Tunisia; b Department of Gastroenterology, Monastir University Hospital, Monastir, Tunisia; c Department of Cardiovascular Surgery, Monastir University Hospital, Monastir, Tunisia; d Department of Anesthesiology B, Monastir University Hospital, Monastir, Tunisia; e Department of Surgery, Sidi Bouzid Hospital, Sidi Bouzid, Tunisia

**Keywords:** Hydatid cyst, Peritoneal rupture, Anaphylactic shock, Peritonitis, Echinococcus granulosus, Case report

## Abstract

The rupture of hepatic hydatid cysts into the peritoneal cavity is an uncommon but life-threatening condition that can result in complications such as peritonitis and anaphylactic shock. This report describes the case of a 39-year-old diabetic male who presented with acute epigastric pain, fever (39°C), and abdominal rigidity. Imaging studies revealed pneumoperitoneum and the rupture of a large hepatic hydatid cyst measuring 20 × 30 cm located in the right lobe of the liver. Emergency laparotomy confirmed the rupture, and surgical management involved peritoneal lavage and initiation of antiparasitic therapy. The patient recovered fully and was discharged 23 days after surgery. Rupture of hepatic hydatid cysts occurs in only 1%-2% of cases and is often associated with large cyst size (>10 cm) and superficial location. Early imaging, particularly with CT, is crucial for diagnosis, while surgical intervention aims to manage the rupture, prevent further complications, and address residual cysts. This case highlights the significance of prompt diagnosis and management to avoid severe outcomes such as secondary peritoneal hydatidosis and anaphylactic shock.

## Introduction

Hydatid cyst, or hydatidosis, is a worldwide disease caused by the larval stage of the Echinococcus granulosus tapeworm [1]. It is endemic in Tunisia. The main complications of hydatid cysts include superinfection and rupture in the bile ducts [2,3]. Furthermore, rupture in the abdominal cavity is a rare complication that represents approximately 1% of cases [4]. It constitutes a true emergency and marks a critical and unfavorable turning point in the progression of hydatidosis, due to the immediate risk of anaphylactic shock and secondary peritoneal hydatidosis. We will focus on this condition following the SCARE criteria [3].

## Case presentation

**Fig. 1:**
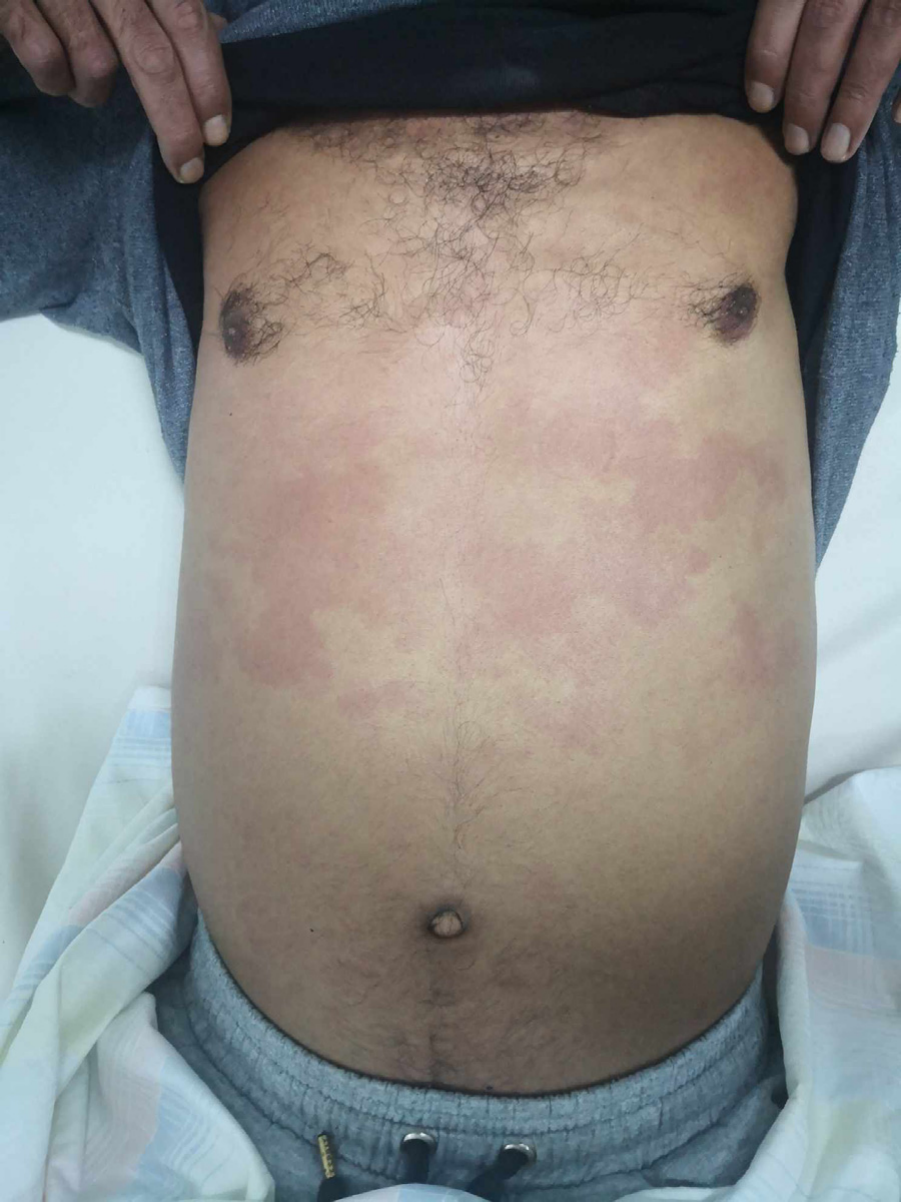
Erythematous lesions occupying the supraumbilical region suggesting the anaphylactic reaction after a rupture of a hydatid cyst.

**Fig. 2:**
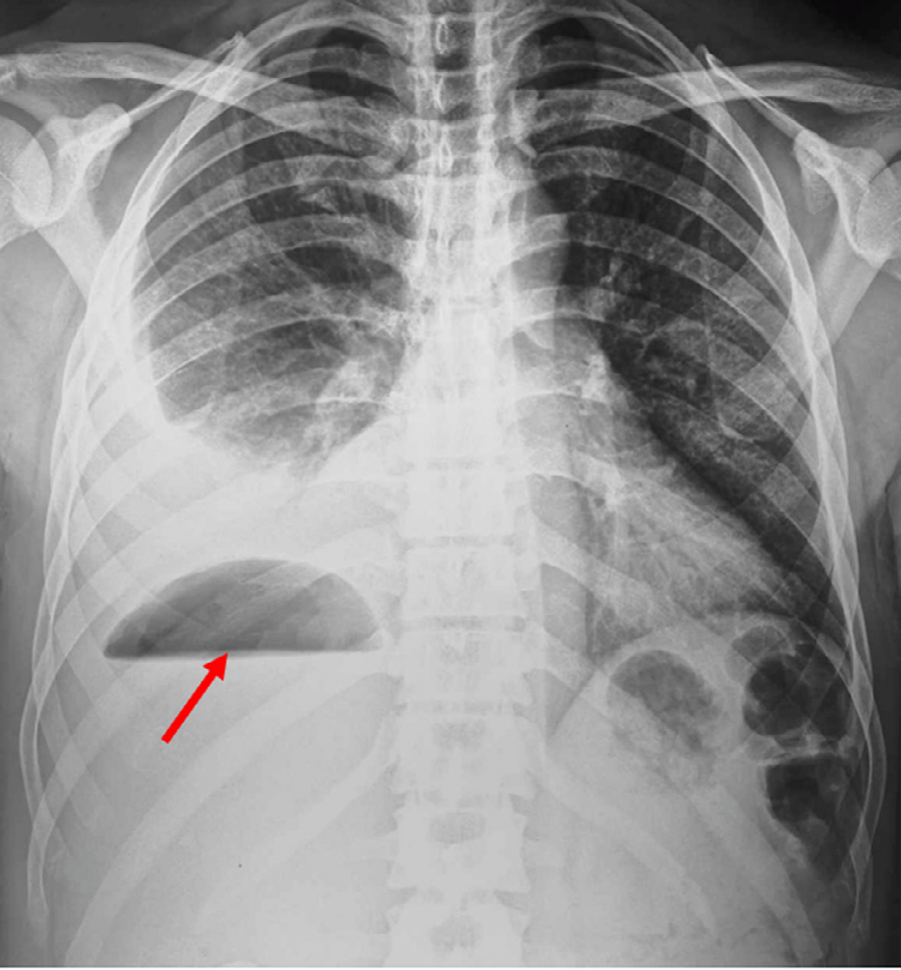
Chest and abdominal X-ray showing the hydro-aeric level in the upper right quadrum of the abdomen (red arrow).

**Fig. 3:**
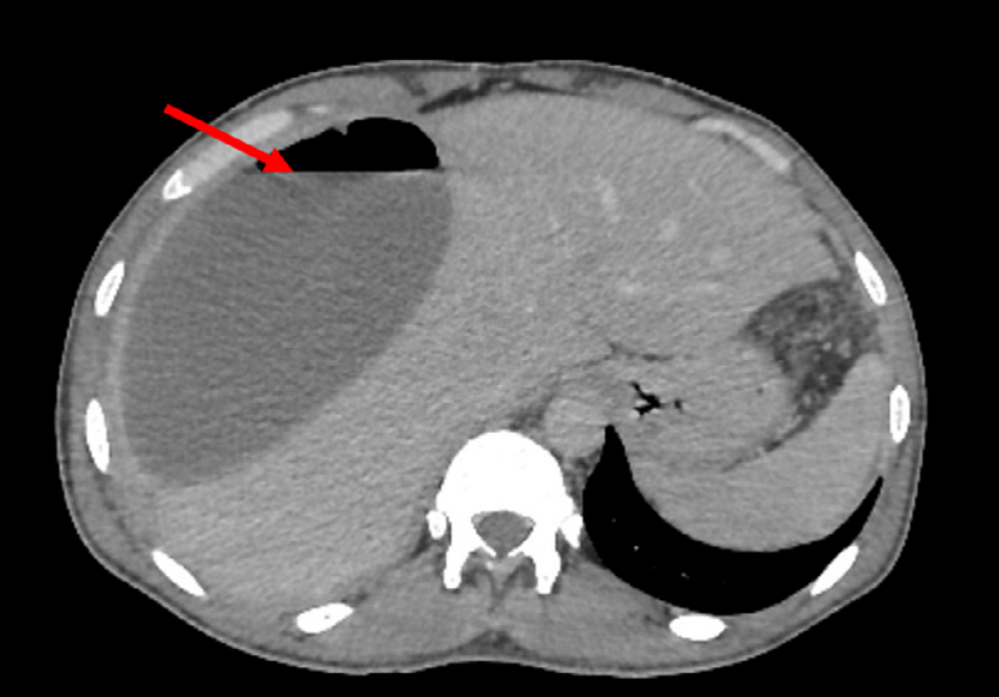
An axial slice of a CT image showing a hepatic abscess with a hydro-aeric level in the upper right quadrum of the abdomen (red arrow).

**Fig. 4:**
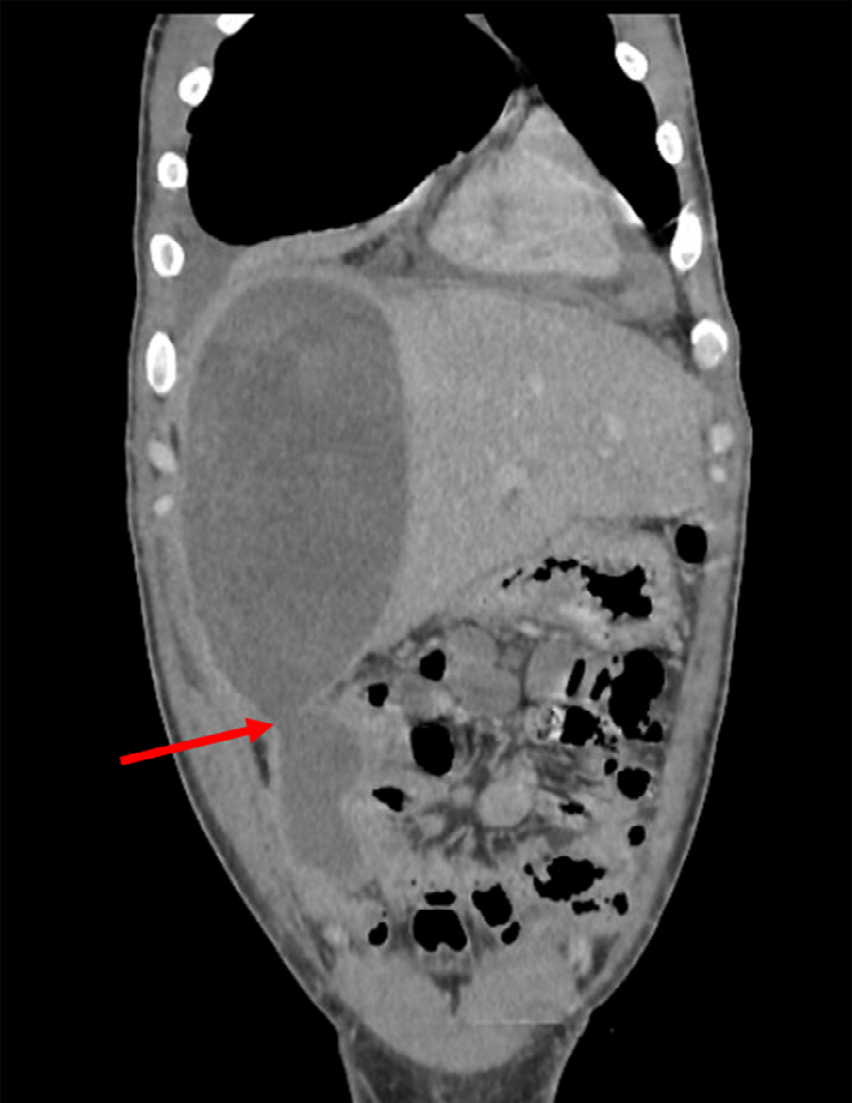
A coronal slice of a CT scan showing the rupture of the huge right hydatid cyst in the peritoneal cavity (red arrow).

A 39-year-old man living in a rural area in North Africa, with a history of type 2 diabetes mellitus, was hospitalized in the urology department for pyelonephritis and treated with antibiotic therapy 2 weeks before current symptoms. Five days before his recent admission, the patient experienced epigastric pain accompanied by a high fever that reached 39.5°C. His epigastric pain intensified significantly 4 hours before hospitalization. Upon admission to the emergency department, a physical examination revealed a high temperature of 39°C and generalized rigidity of the abdominal wall, suggesting peritonitis. In addition, erythematous lesions were observed in the supraumbilical region (Fig. 1). The erythematous changes observed in the supraumbilical region are likely related to the inflammatory response caused by peritoneal irritation due to the rupture of the hepatic hydatid cyst. This localized inflammatory reaction may have been exacerbated by the immune response to the spillage of hydatid material into the peritoneal cavity, a hallmark of this rare complication. The patient's heart rate was 109/min, and his blood pressure was 120/80 mmHg. No clinical signs of anemia or jaundice were evident. Laboratory tests showed a white blood cell count of 19.1 × 10^9^/L with 85.3% neutrophils. Other results included: serum bilirubin, 37.6 μmol/L; aspartate aminotransferase, 51 U/L; alanine aminotransferase, 87 U/L; and C-reactive protein at 300 mg/L. Chest and abdominal x-rays revealed bilateral subdiaphragmatic air, indicative of pneumoperitoneum (Fig. 2). An abdominal ultrasound detected free intraperitoneal air and fluid, but evaluation of liver tissue was impeded by intraperitoneal air, and hypoechoic foci were not observed in the liver. Subsequently, abdominal CT identified a Hydatid cyst of the liver in segments VII and VIII that has ruptured into the peritoneum and extends into the perirenal space (Fig. 3, Fig. 4). The patient was diagnosed with Peritonitis secondary to the rupture of a hepatic hydatid cyst. Brief medical resuscitation was initiated, involving intravenous antibiotic therapy, the use of vasoactive drugs, and adequate fluid resuscitation. An emergency laparotomy was performed. Despite a history of duodenal ulcer, no gastrointestinal perforation was found. Upon discovering, a 20 cm × 30 cm hydatic cyst in the right lobe of the liver. It was ruptured into the peritoneal cavity, spreading the hydatid material throughout. Accordingly, an extensive peritoneal lavage was performed using a hypertonic saline solution, along with resection of the protruding dome, removal of the germinative membrane, and broad drainage. There was a cysto-biliary communication that was sutured. An external drainage was performed. An antiparasitic, Albendazole (400 mg per day), treatment was administered. The patient recovered fully without complications and was discharged from home 23 days after surgery.

## Discussion

Hydatid cyst disease is caused by the parasite *Echinococcus granulosus*, among others. The main species responsible for human infection include *E. granulosus* (leading to cystic echinococcosis), *E. multilocularis* (causing alveolar echinococcosis), *E. vogeli*, and *E. oligarthus*. In this parasitic cycle, dogs are the primary hosts, whereas animals like sheep and pigs act as intermediate hosts. Humans become accidental hosts when they ingest *E. granulosus* eggs, either through contaminated vegetables or by close contact with infected pets [5,6]. The rupture of a hepatic hydatid cyst in the peritoneal cavity is rare. Its incidence ranges from 1% to 2%, according to various studies in the literature [4]. The rupture of a hydatid cyst in the peritoneal cavity is secondary to abdominal trauma or can occur spontaneously. In this case, the CT findings were indicative of a ruptured hepatic hydatid cyst, with pneumoperitoneum suggesting perforation into the peritoneal cavity. The rupture was further evidenced by the spread of hydatid material into the peritoneal and perirenal spaces, a hallmark of hydatid cyst rupture. Certain factors predispose to the rupture of a liver hydatid cyst, notably the young age of the patient, a cyst diameter greater than 10 cm, and the superficial location of the cyst [5,7]. The clinical presentation varies, but 2 common forms are often described: the rupture can develop insidiously, disappearing unnoticed, and result in diffuse secondary peritoneal echinococcosis, or it can present more dramatically, producing a picture of peritonitis with or without anaphylactic shock [8]. In some cases, such as the present one, localized erythematous skin lesions can be observed, which likely result from the severe inflammatory cascade and immune response triggered by hydatid material spillage. Although not commonly described in hydatid cyst rupture, these findings may serve as an additional clinical clue to the severity of the condition, highlighting the urgency of prompt diagnosis and management. The diagnosis is based primarily on abdominal CT scans; in addition, abdominal ultrasound can aid in the diagnosis. CT is crucial for diagnosing hepatic hydatid cysts, providing detailed information on cyst morphology, location, and complications. Typical features include well-defined cystic lesions with internal septations or daughter cysts, peripheral calcifications, and a characteristic water-lily sign representing detached germinal membranes. Urgent surgical intervention is required, usually performed by laparotomy before any germinal membrane occurs. CT imaging also aids in assessing complications, such as secondary peritonitis or abscess formation. The identification of free air and fluid in the peritoneal cavity in this patient highlights the utility of CT in identifying emergent complications of hydatid disease. The surgical approach includes 3 main steps: managing the pericyst, carefully extracting the intact germinal membrane along with its contents a thorough peritoneal lavage. A conservative approach is applied to the pericyst in all cases because of its typically thin and flexible nature. This meticulous procedure minimizes the risk of spillage and additional complications, ensuring optimal management of the condition [9].

## Conclusion

The rupture of a hepatic hydatid cyst in the peritoneal cavity is a rare but serious complication that requires immediate medical attention due to the risk of anaphylactic shock and widespread secondary peritoneal echinococcosis. Early diagnosis by imaging, primarily abdominal CT scans, is critical for timely intervention. Surgical management, typically by laparotomy, aims to carefully remove cyst contents without rupturing the germinal membrane, in conjunction with thorough peritoneal lavage. Adopting a conservative approach to pericyst treatment is crucial, given its thin and flexible nature, to reduce the risk of further complications. Prompt and meticulous surgical intervention, paired with antiparasitic treatment, ensures favorable outcomes and minimizes the risk of recurrence or additional complications in affected patients.

## Ethical approval

Ethical approval is exempt/waived at our institution.

## Research registration number

Not applicable.

## Provenance and peer review

Not commissioned, externally peer reviewed.

## Patient consent

Written informed consent was obtained from the patient for publication of this case report and accompanying images. A copy of the written consent is available for review by the Editor-in-Chief of this journal on request.
